# Licorice Extract Supplementation Affects Antioxidant Activity, Growth-Related Genes, Lipid Metabolism, and Immune Markers in Broiler Chickens

**DOI:** 10.3390/life12060914

**Published:** 2022-06-17

**Authors:** Magda I. Abo-Samaha, Youssef S. Alghamdi, Set A. El-Shobokshy, Sarah Albogami, Eman M. Abd El-Maksoud, Foad Farrag, Mohamed M. Soliman, Mustafa Shukry, Mohamed E. Abd El-Hack

**Affiliations:** 1 Poultry Breeding and Production, Animal Husbandry and Animal Wealth Development, Faculty of Veterinary Medicine, Alexandria University, Edfina 22758, Egypt; magda.abosamaha@alexu.edu.eg; 2 Department of Biology, Turabah University College, Taif University, P.O. Box 11099, Taif 21995, Saudi Arabia; ysghamdi@tu.edu.sa; 3 Department of Nutrition and Veterinary Clinical Nutrition, Faculty of Veterinary Medicine, Alexandria University, Edfina 22758, Egypt; set.abdelsalam@alexu.edu.eg; 4 Department of Biotechnology, College of Science, Taif University, P.O. Box 11099, Taif 21944, Saudi Arabia; dr.sarah@tu.edu.sa; 5 Department of Biochemistry, Faculty of Veterinary Medicine, Alexandria University, Edfina 22758, Egypt; eman.abdelmaksoud@alexu.edu.eg; 6 Department of Anatomy and Embryology, Faculty of Veterinary Medicine, Kafrelsheikh University, Kafrelsheikh 33516, Egypt; foad.farag@keu.edu.eg; 7 Clinical Laboratory Sciences Department, Turabah University College, Taif University, P.O. Box 11099, Taif 21944, Saudi Arabia; mmsoliman@tu.edu.sa; 8 Department of Physiology, Faculty of Veterinary Medicine, Kafrelsheikh University, Kafrelsheikh 33516, Egypt; 9 Poultry Department, Faculty of Agriculture, Zagazig University, Zagazig 44511, Egypt

**Keywords:** broiler, growth performance, blood biochemistry, *Glycyrrhiza glabra*, immunity, gene expression

## Abstract

The objective of this study was to evaluate the *Glycyrrhiza glabra* effect on growth performance, blood parameters, antioxidant and lysosomal activity, histology and immunohistochemistry of liver and intestine, and the gene expression profile of broiler chickens. A total of 180 Cobb500 broiler chicks (one-week-old) were used in this study. Chicks were distributed randomly into three treatment groups; the first group received drinking water without any supplementation (control group). In contrast, birds in groups 2 and 3 received licorice supplementation in drinking water with 0.4 and 0.8 g licorice/liter, respectively. Results revealed that licorice at a 0.4 g/L of water level improved body weight, weight gain, feed intake, and FCR. Licorice also exhibits a broad range of biological activities such as hypolipidemic, hypoglycemic, hepatoprotective, immunostimulant, and antioxidant effects. The morphometric analysis of different parameters of the intestine revealed a significant increase in the intestinal villi length, width, and villi length/crypt depth in the group supplemented with licorice 0.4 gm/L compared to other groups. The number of CD3 positive in both duodenum and ileum was increased in the licorice 0.4 gm/L group compared to other groups. The expression of growth-related genes was significantly increased with licorice supplementation and modulation of the lipid metabolism genes in the liver and upregulated to the mRNA expression of both superoxide dismutase (SOD1) and Catalase (CAT). Our results revealed that licorice supplementation increased the growth performance of broiler chickens and impacted the birds’ antioxidant activity through modulation of the growth-related genes, lipid metabolic markers, and antioxidant-related pathways.

## 1. Introduction

In the last few decades, antibiotics have been applied to maintain health and productivity in the poultry industry. On the other hand, antibiotic growth promoters are banned in many countries due to increasing concerns about the transmission and proliferation of resistant strains through the food chain [1]. As a result, the investigation for alternatives to antibiotics, such as medicinal herbs, has gained an interest in poultry nutrition in recent years. Medicinal plant extracts may improve poultry development performance by increasing digestibility, stimulating digestive enzyme release, preventing tissue oxidation, and modulating gut bacteria populations [2].

Natural feed additives based on bioactive components from herbal plants have a lot of potential. Nutritional supplements help chickens grow, feed efficiency, nutrient digestion, and antioxidant status. Licorice has been used in various herbal formulae in several pieces of research. Flavonoids and glycyrrhizin, two bioactive components, can be found in licorice. There are numerous pharmacological qualities in the roots of this herb that include antioxidant, antiviral, antibacterial, and anti-inflammatory effects. An immune system, liver, and lung disease treatment can benefit from the use of licorice extracts (LE) [3].

For almost 4000 years, the roots of licorice have been utilized as a medicinal substance [4]. The flavonoid components hispaglabridins and glabridins give the licorice root its yellowish color [4]. Glycyrrhizinic acid is found in roughly 4–25% of dried aqueous extracts of licorice [5]. Liquiritin, isoliquiritigenin, liquiritigenin, glycyrrhetinic acid, and glycyrrhiza polysaccharide are the active components in licorice. The herb has abundant flavonoids and syringic, abscisic, trans-ferulic, 2,5-dihydroxy benzoic, abscisic, and salicylic acids [6,7]. Licorice has antimicrobial [8], anti-atherosclerotic and estrogenic [9], anti-nephritic, and antioxidant effects, among others [10]. The components glycyrrhizin, glycyrrhizinic acid, glebridine, apigenin, and liqualkone have already been antimicrobial, antioxidant, and anti-inflammatory properties [9].

Supplementation in poultry diets significantly impacts their productive performance by improving organ growth, accelerating digestion, and promoting hunger. Other health benefits include cleansing, anti-bacterial, and anti-inflammatory properties in addition to its growth-promoting properties [3]

In a previous study, Sedghi et al. [11] discovered that adding LE to broiler diets (0.5, 1, or 2 g/kg) had no effect on body weights or feed efficiency but that adding licorice to broiler diets reduced abdominal fat content and serum cholesterol and low-density lipoprotein-cholesterol concentrations when compared to the control. Another study found that using *G. glabra* extract powder at 0.25, 0.5, and 1 g/kg feed increased broiler chicken growth over 35 days [12]. The use of LE in chicken drinking water at a concentration of up to 0.4 g/L enhanced feed intake and improved immune response, antioxidant, and lipid profiles [13]. Broiler hens’ body gain and feed efficiency improved when licorice root extracts were added to their diet [14]. As a result, licorice has been suggested as an alternative to antibiotics as a growth booster in broiler chickens [15].

Licorice has been found to have a wide range of favorable health effects, including immunomodulatory, anti-bacterial, antioxidative, anti-inflammatory, anti-diabetic, hepatoprotective, antiviral, anti-infective, and radical-scavenging properties [6,7]. Licorice root extract was shown to contain a variety of phytochemicals, including flavonoids (isoflavonoids, formononetin, and liquiritin), saponin triterpenes (liquirtic acid, and glycyrrhizin), sugars, coumarins, amino acids, starch, tannins, phytosterols, choline, and several vitamins (such as ascorbic acid) [6]. Previous investigations revealed that licorice contains 20 triterpenoids and 300 flavonoids [16]. The licorice root extract contains up to 25% glycyrrhizin [17]. Glycyrrhizin contains glucuronic acid (2 molecules) and glycyrrhetinic acid (1 molecule) [18]. The chemically examined licorice revealed its nutritional breakdown: carbohydrate (47.11%), fibre (24.48%), protein (9.15%), silica (3.56%), and low-fat content (0.53%) [19].

This root was also discovered to have 7.70% of its mass in ash and 6.80% in moisture. Calcium and phosphorus concentration in raw LE was reported to be 1720 and 78 mg/100 g, respectively. The principal amino acid components discovered in LE were proline (1.02%), aspartic (0.88%), alanine (0.51%), and glutamic acid (0.51%) [19].

Due to the presence of flavonoid components such as hispaglabridins and glabridins, the color of licorice root is yellow [20]. The dried aqueous extracts of licorice, on the other hand, contain between 4 and 25 percent of glycyrrhizinic acid [5]. In addition to glycyrrhetinic acid and glycyrrhiza polysaccharide as the primary active components, licorice contains liquiritin, isoliquiritigenin, and liquiritigenin, as well as flavonoids and the syringic, abscisic, trans-ferrulic, 2,5-dihydroxy benzoic, abscis. Glycyrrhizin, 18-glycyrrhetinic acid, glabrin A and B, and isoflavones of *Glycyrrhiza glabra* Linn. have pharmacological effects [6].

The impacts of licorice on body weight gain of broilers in association with different blood parameters, intestinal histopathology, and gene expression are not fully known. Therefore, the objective of the present study is to characterize the effects of licorice supplementation in drinking water, as a potential antimicrobial agent, on production performance, carcass quality, blood parameters, intestinal histopathology, and gene expression in broiler chickens. This study examines the pathway by which the licorice induced its beneficial effects.

## 2. Materials and Methods

### 2.1. Animal Care

The Ethics Committee of the Local Experimental Animals Care Committee accepted the study, which was carried out following the Institutional Animal Care and Use Committee of the University of Alexandria approved this study’s experimental protocol (Permit #2022/013/111).

### 2.2. Experimental Design

A total of 180 Cobb500 broiler chicks (one week old) were used in this study. Chicks were distributed randomly into three treatment groups (60 chicks/treatment) with three replicates per treatment (20 chicks per replicate). The control group received drinking water without any supplementation. In contrast, birds belonging to the remaining groups 2 and 3 received licorice supplementation in drinking water with 0.4 and 0.8 g/liter, respectively. Licorice was obtained from a commercial company (Sekem Group, Cairo, Egypt). Aqueous licorice solutions were prepared by soaking two different amounts (0.4 and 0.8 g) of licorice root powder in a liter of water; after that, the solutions were mixed for 24 h at room temperature in a mixer before being filtered using Whatman No. 42 filter sheets (Hawach Scientific Co., Ltd., Xi’an, China) The solutions were used for the experiment, according to [21]. The supplementation of licorice in the drinking water started at one week old and lasted till 42 days, and the solution was added to whole day water intake. The chicks were reared on the floor in an environmentally controlled room. The temperature regimen gradually decreased from 32 to 24 °C by 3.5 °C weekly. A continuous lighting program was provided on the first day of arrival, followed by a 23 L:1 D lighting schedule. According to the National Research Council, all groups received a basal diet shown in Table 1 [22]. Broiler chicks were fed on starter, grower, and finisher diets during the first two weeks, 3–4 weeks, and 5–6 weeks of the experiment, respectively. The ingredient composition and chemical analysis of the basal diets used for the starter, grower, and finisher periods is presented in Table 1.

### 2.3. Chemical Analysis of Ration: Dry Matter and Crude Nutrients

Dry matter contents of feed samples were determined by oven-drying at 105 °C for eight hours [23]. Ash contents were determined by incineration at 550 °C overnight. Crude protein was determined by using the Kjeldahl method according to [24], and ether extract was determined according to [25].

### 2.4. Growth Performance Parameters

Birds were individually weighed every week starting from one week old age till 6th week of age. Feed intake was calculated weekly for each group, and body weight gain was calculated as the difference between two successive weights. (FCR) was calculated by dividing the amount of feed consumed (g) during the week by the bodyweight gain (g) during the same week.

### 2.5. Sample Collection

At the end of the experiment (42 d), Blood samples were collected by brachial vein puncture into plane vacuum tubes and centrifuged at 2500× *g* for 10 min. Serum was aspirated and located in a 2.5 mL centrifuge tube and stored at −20 °C until analysis for malondialdehyde (MDA), lysosomal activity, GSH, CAT, lipid profile, liver function tests, and glucose. The liver was collected immediately and divided into two parts; one was kept at −80 °C and used for gene expression analyses. The second part was preserved in 10% neutral formalin with the duodenum, ileum, and cecum for histopathological and immunohistochemistry examination.

### 2.6. Relative Organs Weight

For relative organ weight, nine birds from each group were slaughtered. The liver, gizzard, proventriculus, heart, spleen, thymus, bursa of Fabricius, intestine, and abdominal fat were weighed and recorded relative to body weight.

### 2.7. Chemical Analysis of Serum Samples

An enzymatic method was utilized to assess the total serum cholesterol TC using colorimetric diagnostic kits (Vitro Scient kits, Cairo, Egypt) following the procedures of Allain et al. [26]. The enzymatic colorimetric method was used to determine the serum level of high-density lipoprotein (HDL)-C following the procedures of Vassault et al. [27]. The Iranian formula of low-density lipoprotein (LDL)-C = total cholesterol (TC)/1.19 + triglycerides (TG)/1.9 − HDL/1.1 − 38 was used for LDL-C calculation. A peroxidase-coupled assay was utilized for the colorimetric determination of serum triglycerides following the practices of McGowan et al. [28] using Vitro Scient kits. FFA, TG, total protein, albumin, glucose levels, ALT, and AST activities were analyzed using commercially available enzymatic spectrophotometric kits (Vitro Scient kits, Egypt).

### 2.8. Antioxidant and Lysosomal Activity in Blood

Estimation of serum malondialdehyde (MDA), catalase, and reduced glutathione (GSH) was made colorimetrically by using a microplate spectrophotometer with a commercial detection kit (Bio-Diagnostic, Giza, Egypt) following the manufacturer’s instruction. Lysozyme was estimated using kits from Elabscience Biotechnology Co., Ltd. (Houston, TX, USA) in an enzyme-linked immunosorbent assay (ELISA) reader following the manufacturer’s instructions [29]. Briefly, 10 mL of serum was combined with 190 mL of a solution containing 0.2 mg of *Micrococcus lysodeikticus* per mL of PSB, pH = 7.4; 1 to 5 min at room temperature was spent shaking the plate. Using a microplate reader, a microplate reader was measured at 450 nm after each time point (UVM). A unit of lysozyme activity is the amount of enzyme providing a 0.001/min absorption reduction.

### 2.9. Gene Expression Analysis

According to the manufacturer’s instructions, total RNA was isolated with the TRIzol reagent (Life Technologies, Gaithersburg, MD, USA). cDNA was instantly equipped using the MultiScribe RT enzyme kit (Applied Biosystems, Foster City, CA, USA). The resultant cDNA was exposed in triplicate for real-time PCR analysis. Real-time PCR was achieved using Power SYBR Green PCR Master Mix (Applied Biosystems, Life Technologies, CA, USA) on a 7500 Real-Time PCR Systems (Applied Biosystems, Foster City, CA, USA). Compared to control, the relative fold change in mRNA expression for each gene under study, namely, catalase, superoxidismutase1, insulin growth factor-1, growth hormone receptor, peroxisome proliferator-activated receptor, fatty acid synthase, and lipoprotein lipase, was calculated. The fold change in mRNA expression of the genes tested was normalized using the expression of the housekeeping gene—B.actin. Table 2 provides the primer sequences and accession numbers for the genes.

### 2.10. Histologic Staining and Immunohistochemistry

Tissue samples from the intestine (duodenum, ileum, and cecum) and liver were collected and fixed by immersion in 4% buffered paraformaldehyde. After fixation for 24 h at room temperature (25 °C), the tissues were dehydrated with ascending concentrations of ethyl alcohol, embedded in paraffin, cut into 3–5 μm sections with a microtome (RM2135; Leica, Munich, Germany), and stained with hematoxylin and eosin for general histological and morphometric examination.

For a demonstration of T lymphocytes, sections were also collected to perform cluster of differentiation 3 (CD3) immunohistochemistry. Immunoperoxidase staining was performed as described by [30] with few modifications. Briefly, tissue sections were sectioned at 5 µm on pre-coated glass slides. The endogenous peroxidase block was then applied for 5 min. Antigen retrieval was performed by boiling the sections for 20 min in citrate-based buffer (Dako target retrieval solution, pH 6, Agilent Technologies, Santa Clara, CA, USA). The non-specific reaction was blocked by 0.2% bovine serum albumin. The sections were incubated for 30 min at room temperature with anti-human CD3 rabbit polyclonal antibody (Dako; ready-to-use). The diluted biotinylated rabbit anti-mouse secondary antibody (Dako A/S, Glostrup, Denmark) was used for 30 min in a humidified box at room temperature. The sections were rinsed in 0.01 M PBS, pH 7.6. A substrate, 0.5 mg 3,3-diaminobenzidine-tetrahydrochloride (DAB, Sigma, St. Louis, MO, USA) per mL Tris ± HCl buffer (0.05 M, pH 7.6) containing 0.01% H_2_O_2_ was used as a chromogen. The slides were counter-stained with hematoxylin, rinsed in tap water, dehydrated in alcohol, then xylene, and mounted in DPX (BDH, Poole, UK). Control slides were incubated as described above, except that the primary antibodies were omitted.

### 2.11. Statistical Analysis

The statistical analysis was performed using SAS [31]. One-way analysis of variance (ANOVA) with subsequent Duncan’s post hoc test was used for analyses. The overall significance level was set as *p* < 0.05, *p* < 0.01, and *p* < 0.001. All values are expressed as the mean ± standard error. Statistical model:

X_ij_ = μ + T_i_ + e_ij_

where X_ij_ = Value of ith observation (the variable as body weight) of the ith treatment, μ = Overall mean, T_i_ = Effect of ith treatment (treatment: three different treatments), and e_ij_ = Random error.

## 3. Results

### 3.1. Growth Performance

Bodyweight (BW), body weight gain, feed intake, and feed conversion ratio were presented in Table 3. We observed that BW at the second and third weeks was significantly different, with groups 2 and 3 BW greater than (the control group). For the fourth week, group 2 was higher than groups 1 and 3, with no significant difference between groups 1 and 3. In the fifth week, BW was most increased for group 2 (2360.6 g), followed by group 3 (2230.1 g), then group 1 (2078.57 g). The final BW (6th week of age) group 2 was markedly (*p* < 0.0001) higher than group 3 and group 1 by 227.53 and 306.5 g, respectively. Similarly, weight gain was significantly different among groups, with the highest gain recorded for group 2 followed by group 3. The minor gain was recorded for (control). Licorice also increased feed intake in groups 2 and 3 compared to the control group. The average FCR was improved in group 2 (birds received 0.4 g/L water), where FCR was the least among groups, followed by the control group, while the highest FCR was recorded for group 3 (birds received 0.8 g/L water).

### 3.2. Relative Organs Weight

Data on the effect of licorice supplementation in drinking water on some organs’ relative weight of broiler chicken are presented in Table 4. There were no significant differences among liver, gizzard, proventriculus, heart, spleen, thymus, and relative intestine weight. The abdominal fat percentage decreased significantly with licorice supplementation in group 2 compared to the control group; with increasing the amount of licorice in drinking water, the abdominal fat also reduced in group 3 compared to group 2. Bursa of Fabricius was significantly different among groups, with group 2 having the most significant value compared to group 3 and the control group.

### 3.3. Serum Lipid Profile

Data on the effects of two licorice treatments on the serum lipid profile of 42 days old broilers are given in Table 5. Licorice supplementation significantly increased serum HDL-C concentration and HDL-C/LDL-C ratio compared with the control group. Moreover, licorice significantly (*p* < 0.0001) decreased total cholesterol (TC), low-density lipoprotein cholesterol (LDL-C), free fatty acid (FFA), and triglyceride (TG) levels.

### 3.4. Serum Liver Biomarker and Serum Glucose Level

Table 6 shows that in a comparison to control values, supplementation with two licorice (0.4 g/L and 0.8 g/L) treatments induced a significant (*p* < 0.0001) reduction in serum ALT AST activity, confirming the hepatoprotective effect of licorice. Furthermore, treatment with licorice 0.8 g/L substantially reduces ALT activity more than the other treatment, alongside a significant (*p* < 0.0001) reduction in serum glucose level, confirming its hypoglycemic effect. The result showed insignificant differences in serum proteins (total protein, albumin, and globulin) among the treatment.

### 3.5. Oxidative Stress, Immunostimulant Biomarkers

Table 7 showed that in comparison to the control group, supplementation with two licorice (0.4 g/L and 0.8 g/L) treatments inhibited the lipid peroxidation of mitochondria and decreased the oxidative rate, which was evidenced by a significant (*p* < 0.0001) reduction in MDA and increment in GSH and CAT, which indicate the antioxidant effect of licorice alongside with a significant (*p* < 0.0001) elevation in lysosomal activity, especially with group 3 confirming its immunomodulatory effect.

### 3.6. Histologic Staining and Immunohistochemistry

**Figure 1:**
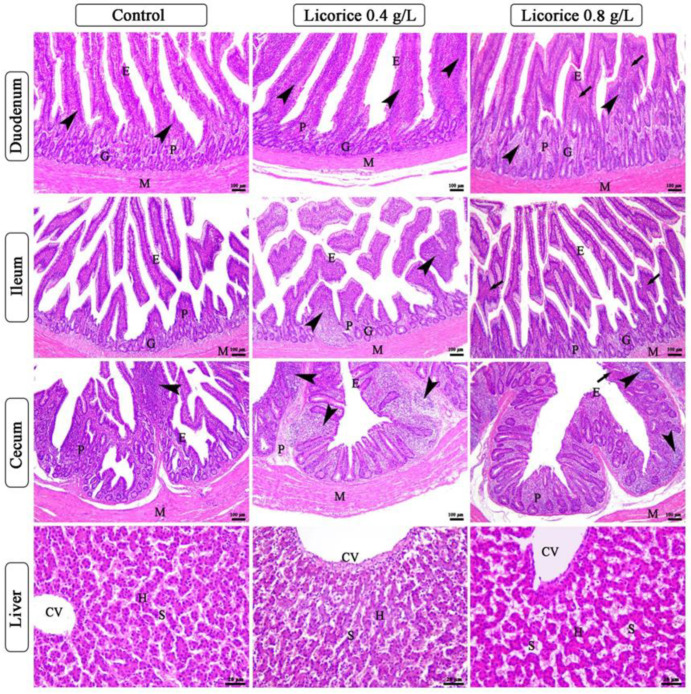
Photomicrograph of duodenum, ileum, cecum, and liver of broiler chickens of control, 0.4 gm/L and 0.8 gm/L licorice acid groups. The panels of the duodenum, ileum, and cecum show simple columnar epithelium of lamina epithelial (E), lamina propria submucosa (P), mucosal glands (G), tunica muscularis (M), lymphatic tissue in both lamina epithelial and lamina propria (arrowheads), congested blood vessels of lamina propria (arrows). The panels of the liver show central vein (CV), hepatocytes (H), and blood sinusoids (S), which appear dilated and congested in the 0.8 gm/L licorice acid group. Stain H&E.

The intestine wall was composed of tunica mucosa, submucosa, muscularis, and serosa. The tunica mucosa comprises lamina epithelial of simple columnar epithelium with goblet cells, lamina propria of loose connective tissue containing diffuse lymphatic tissue, and mucosal glands in the small intestine. The lymphatic tissue is abundant in the cecum forming cecal tonsils in the basal part of the cecum (Figure 1). The histological examination of different parts of the intestine revealed an increase in the lymphatic elements in both laminae epithelial and lamina propria of duodenum and ileum of licorice 0.4 gm/L group compared with a control group and licorice 0.8 gm/L, which shows congestion of blood vessels of lamina propria (Figure 1). The morphometric analysis of different parameters of the intestine revealed a significant increase in the intestinal villi length, width, and villi length/crypt depth in the group supplemented with licorice 0.4 gm/L compared with the other two groups Table 8.

**Figure 2:**
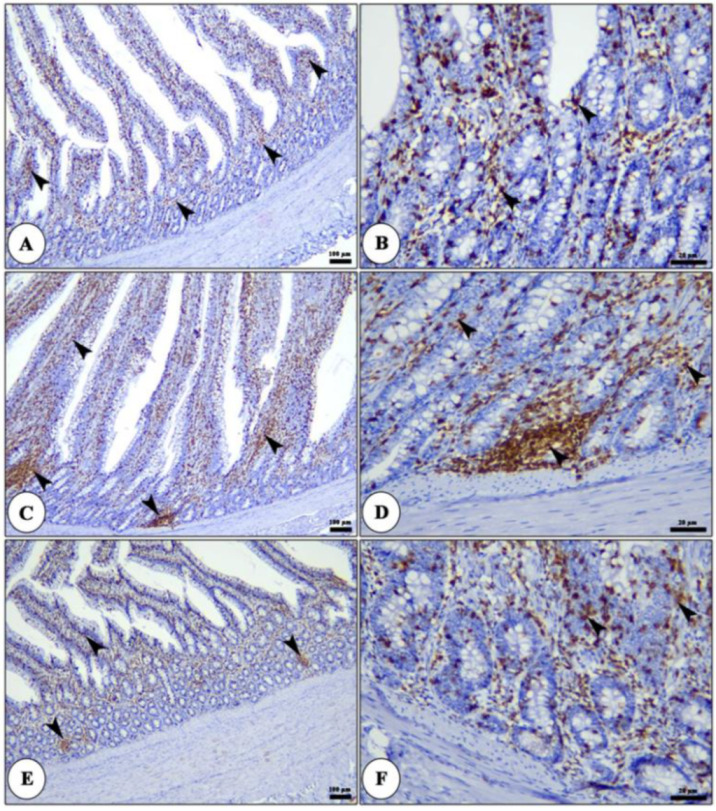
Photomicrograph of CD3 immune staining of duodenum of broiler chickens of control (panel (**A**,**B**)), 0.4 gm/L (panel (**C**,**D**)), and 0.8 gm/L (panel (**E**,**F**)) licorice acid groups showing CD3 +ve cells in the form of diffuse T lymphocytes in the lamina epithelial and lamina propria and/or lymphatic nodules in the lamina propria (arrowheads).

**Figure 3:**
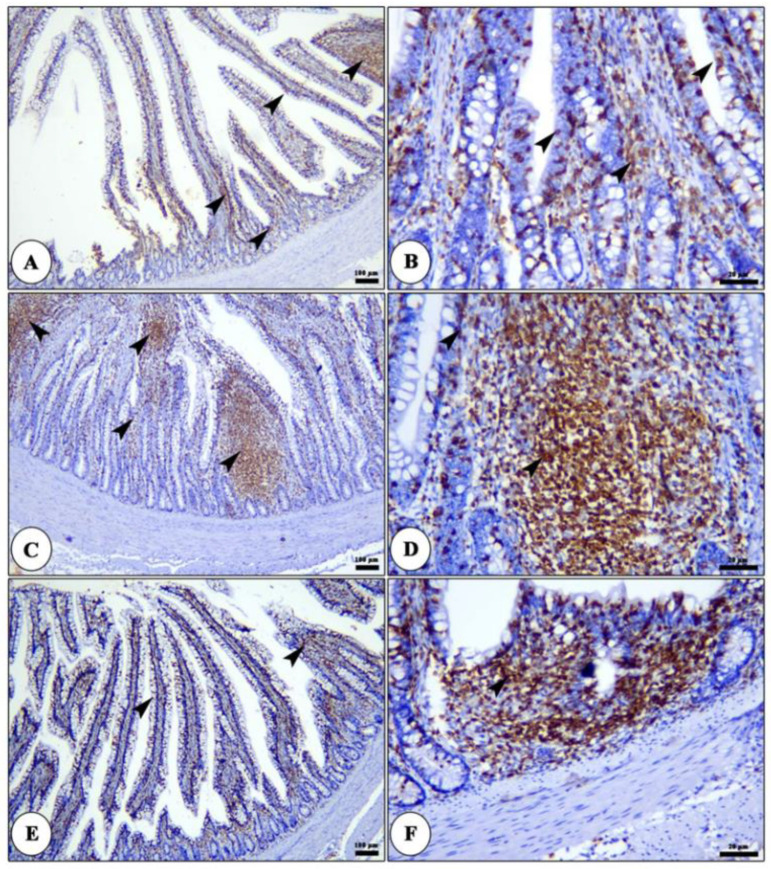
Photomicrograph of CD3 immune staining of ileum of broiler chickens of control (panel (**A**,**B**)), 0.4 gm/L (panel (**C**,**D**)) and 0.8 gm/L (panel (**E**,**F**)) licorice acid groups showing CD3 +ve cells in form of diffuse T. lymphocytes in the lamina epithelialis and lamina propria and/or lymphatic nodules in the lamina propria (arrow heads).

The liver of all groups shows intact polyhedral-shaped hepatocytes radiating from a central vein and separated by hepatic blood sinusoids, which show marked dilatation and congestion in licorice acid 0.8 gm/L (Figure 1). The CD3 positive cells were localized in the lamina propria or migrated in the intestinal epithelium. These cells are arranged in a modular-like structure in the lamina propria or sporadically distributed in both lamina propria and lamina epithelial. The CD3 positive cells increased gradually from the duodenum to the cecum (Figure 2 and Figure 3). The number of CD3 positive in both duodenum and ileum was raised in the licorice 0.4 gm/L group compared with the control and licorice 0.8 gm/L in both lamina propria and lamina epithelial (Figure 2). In contrast, in the cecum, the density of CD3 positive cells shows non-significant changes among the licorice treated groups and control group (Figure 3).

### 3.7. Gene Expression Analysis

**Figure 4:**
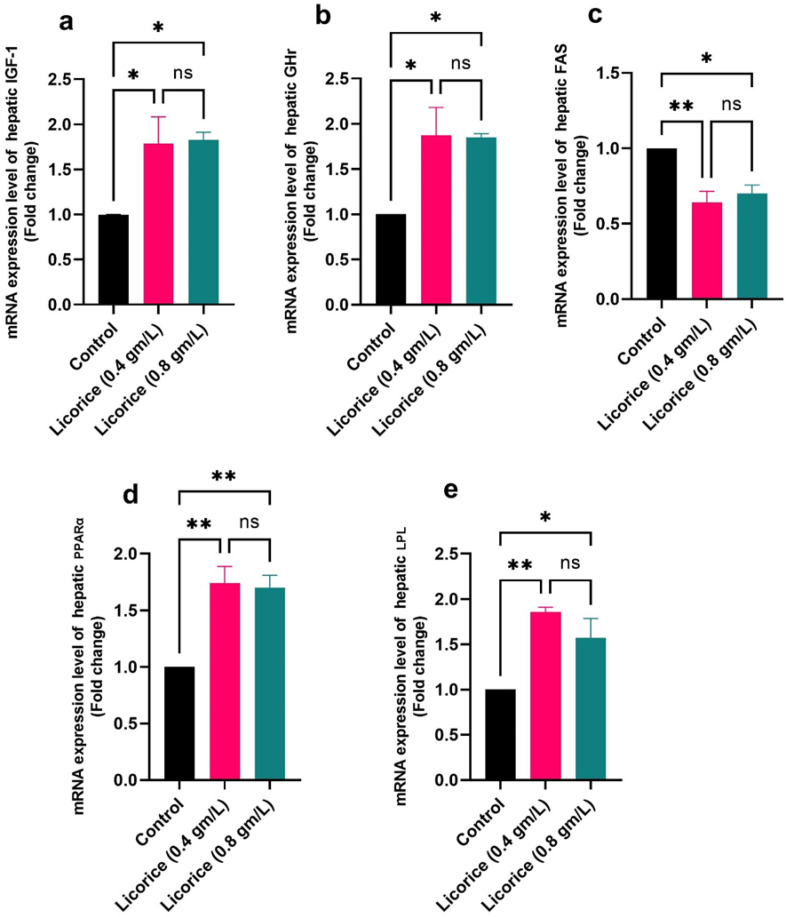
Effect of licorice acid treatment (0.4 and 0.8 gm/L) on hepatic mRNA expression of IGF-1 (**a**), GHr (**b**), FAS (**c**), PPARα (**d**), and LPL (**e**). The data are represented as the mean ± SE. * and ** indicate *p*-value < 0.05, and *p*-value < 0.01 respectively. ns: non-significant. (ANOVA with Dunnett’s multiple comparison test).

**Figure 5:**
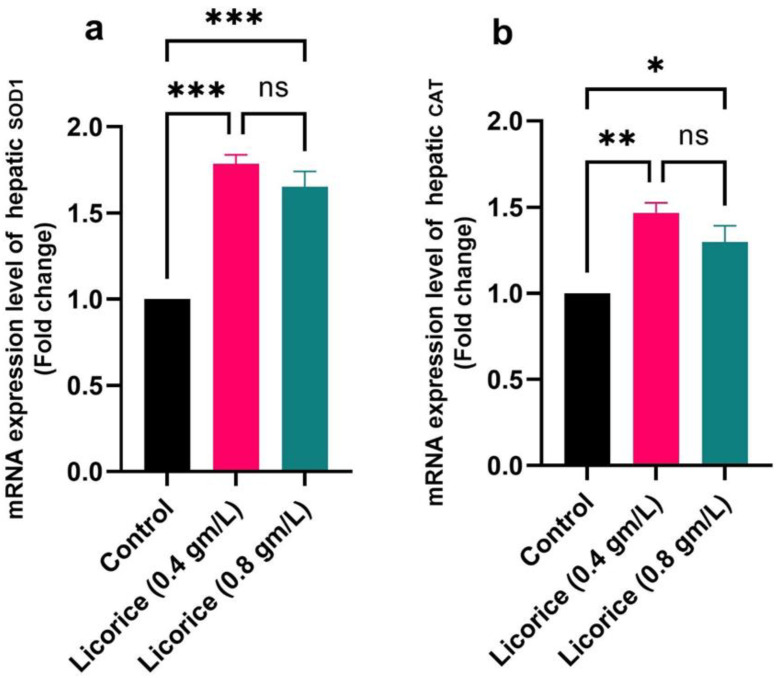
Effect of licorice acid treatment (0.4 and 0.8 gm/L) on hepatic mRNA expression of SOD1 (**a**), CAT (**b**). The data are represented as the mean ± SE. *, ** and *** indicate *p*-value < 0.05, *p*-value < 0.01, and *p*-value < 0.001 respectively. ns: non-significant. (ANOVA with Dunnett’s multiple comparison test).

Figure 4 and Figure 5 show that in comparison to the control group, supplementation with two licorice (0.4 g/L and 0.8 g/L) treatments upregulated the IGF-1, GHr, PPARα, and LPL, with downregulated the mRNA expression of FAS; in addition, the licorice supplemented groups should significantly increase mRNA expression of SOD1 and CAT genes concerning the control group one.

## 4. Discussion

Supplementation of feed additives or growth promoters, which positively impacts their overall health and performance, is frequently used to boost poultry’s growth and laying performance [32,33]. In the present study, birds supplemented with 0.4 g LE/L via drinking water had the highest body weight and weight gain among the treatment groups while having the least FCR. In a similar study, Rashidi et al. [15] found that birds supplemented with LE had the highest BWG in both the grower and total phases when stocking density was increased compared to the control group, supplementing the broiler chickens’ basal diet with 1% LE improved body weight and FCR at 42 days of age [14]. The addition of 200 ppm of licorice root extract with a 1% probiotic supplement to the quail diet boosted the quantity of daily feed intake and body weight gain in Japanese quails [34]. Another study found that supplementing broiler chicks’ diets with garlic and licorice (at 0.25, 0.50, and 1% concentrations) enhanced their productivity [35]. Furthermore, LE improved the performance of heat-stressed broiler chickens in terms of productivity [36]. The addition of *G. glabra* to chickens’ feed improved their growth performance by promoting the development of their organs. The positive effect of licorice supplementation on internal organ development may be one of the reasons behind enhanced poultry growth [3]. The increase in the body weight gain may be due to improving the digestion and appetite in broilers supplemented with licorice [37]. Both animal health and output will benefit from this. licorice contains chemical components that serve as immunomodulators and promote a healthy digestive system [36]. However, not all feeding trials have been positive. Dietary supplementation of licorice significantly of licorice could be associated with its role in improving the metabolism and the immune response under stress conditions. In addition, broilers fed diets enriched with 2.5 g/kg *G. glabra* had better digestion and appetite. Furthermore, including up to 0.5% *G. glabra* in chicken diets during the pullet growth stage improved laying hen performance [38]. When compared to non-treated controls, broilers fed with glycyrrhizic acid (GRA) (60 g/mL in water) had higher body weight gain (BG), final body weight, better FCR, and the lowest mortality rate [37]. According to Sohail et al. [17], dietary antioxidant supplementation can increase avian feed consumption while reducing oxidative damage caused by reactive oxygen substances under stress. In the present study, the highest feed intake was recorded in groups supplemented with licorice compared to the control group. This result was in agreement with Salary et al. [2], who found that introducing 2% licorice extract in drinking water during the starter phase of the rearing period enhanced broiler feed intake by improving feed palatability and stimulating appetite in broilers. Similarly, the addition of 0.4% LE to broiler drinking water enhanced feed consumption (*p* < 0.05) at 21 and 42 days [39]. On the other hand, the feed intake of laying hens fed 0.5, 1.0, 1.5, and 2.0 percent licorice powder added to the basal diet remained unaffected [2]. In a similar study, Hosseini et al. [40] tested a 5 g licorice/kg broiler diet and observed no significant influence (*p* > 0.05) on body weight, feed intake, FCR, livability, and production index. Compared to the control group, Moradi et al. [41] found that adding 0.1, 0.2, and 0.3 mg LE/L drinking water to broiler chicks had no significant influence on their body weight, feed intake, or FCR. Furthermore, when compared to the non-supplemented group, Sedghi et al. [11] used a 0.5, 1, and 2 g LE/kg broiler diet and found no influence on broiler weight, feed intake, or FCR. However, when the licorice was changed and combined, another study found different results. Supplementing feed with licorice as a feed additive improves poultry’s health and growth performance [32]. As a result, laying hens’ performance was improved when broilers were fed diets enriched with licorice acid during the pullet growth stage [13]. There were no significant differences among groups for liver, gizzard, proventriculus, heart, spleen, thymus, and relative intestine weight. This finding was in line with Rashidi et al. [15], who found that including LE in experimental diets had no effect on liver and gizzard percentages during the duration of the investigation. Similarly, Myandoab et al. [34] found that adding licorice root extract and probiotics to Japanese quail diets did not affect carcass percentage or carcass component ratios. Sedghi et al. [11] added LE to broiler diets but found no effect on a carcass weight or internal organs such as the liver, heart, spleen, or bursa. In contrast to Sedghi et al. and Moradi et al. [11,41], in this study, we found that birds receiving 0.4 g/L drinking water had considerably heavier bursa of Fabricius. This could be because licorice root is a potent anti-inflammatory agent that detoxifies and protects the liver, lymphoid organs, and other visceral tissues [42]. This result was disputed by those who claimed that licorice did not influence lymphoid organ relative weight. In this study, licorice supplementation reduced abdominal fat. It was significantly reduced when broilers were given water containing 0.3 g/L of LE. This finding is similar to that of [41], who found that the abdominal fat percentage decreased as the level of LE in drinking water increased. Licorice was also found to reduce abdominal fat in broiler chickens by [11]. Licorice flavonoids also reduced abdominal fat in other species [43,44]. The effect of licorice on the reduction in abdominal fat could be due to suppression in energy intake, reduction in lipid absorption, enhancement of fatty acid oxidation, or reduction in the biosynthesis of fatty acids [44].

Herbal feed additives are among the most important ways to promote growth performance and improve immunological indices, antioxidant status, and poultry health [13]. Hence, we evaluated the effect of licorice supplementation in drinking water with (0.4 g/L and 0.8 g/L) on the growth performance, immunity status, serum antioxidant capacity, and lipid and glucose-lowering effect in broilers. Our study revealed that the addition of licorice in drinking water with (0.4 g/L and 0.8 g/L) has antioxidant activity via a reduction in MDA level and increment in GSH level and CAT activity. Similar results were described by Visavadiya and Narasimhacharya [45], who showed that *Glycyrrhiza glabra* (the primary bioactive component of licorice root) declined hepatic MDA levels with a concomitant elevation in catalase and superoxide dismutase (SOD) activities and total ascorbic acid content. GSH has a significant antioxidant and detoxification role in the liver [46]. Isoliquiritigenin (ISL, a natural active compound of licorice) has been considered a potent antioxidant with an anti-inflammatory effect [47]. Flavonoids (isoflavonoids and liquidity), glycyrrhizic acid, liquiritigenin, triterpenes (glycyrrhizin), and saponins are bioactive components of licorice root. They have anti-inflammatory and antioxidant activities [48,49]. Licorice extracts suppress mitochondrial lipid peroxidation, reduce the oxidative rate and reactive substance generation of thiobarbituric acid, scavenge free radicals, promote antioxidant enzyme activity, and inhibit phospholipase A2, a key enzyme in inflammatory processes. Licochalcone reduces lipopolysaccharide-induced inflammatory reactions; Lico A from licorice root suppresses NF-B and p38/ERK MAPK signaling [50,51]. They analyzed licorice’s antioxidative effects on LDL oxidation. There are seven antioxidative components, including iso-flavans, hispaglabridin A, B, glabridin, and 4*-O-methylglabridin [50,52]. From the previous data, we can conclude that licorice extract has a potential role in improving the antioxidant capacity in broiler chickens. Regarding the antioxidant activities, Hashem et al. [53] revealed that licorice extract has antioxidant activities through elevation of the serum CAT and SOD levels with a significant reduction in MDA level due to its flavonoid content. Moreover, Habibi et al. [54] described that using 0, 7.5, and 15 g/kg of licorice root in broiler chickens declined the MDA level and improved the antioxidant enzyme properties. In addition, Sen et al. [55] reported that licorice extract increased SOD and catalase activities in diabetic mice.

Standard liver function tests are ALT, AST, total protein, albumin, and globulin. AST and ALT activities are most generally biochemical markers for observing chemically prompted liver damage. Hashem et al. [53] approved that the administration of licorice extract to lead-induced oxidative stress in rats has a hepatoprotective effect monitored via suppressing the enzyme activity of ALT and AST. They also reported that rats who received licorice only had non-significant changes in TP, albumin, globulin, and A/G. Furthermore, Glycyrrhizic acid prevents cell membrane permeability changes and increases hepatocytes’ survival rate. These outcomes agreed with our result that revealed that treatment with licorice extract induced a significant (*p* < 0.0001) reduction in serum ALT, AST activity, and insignificant differences in serum proteins (total protein, albumin, and globulin) among the treatments. These data also harmonize with Huo et al. [56], who proved the hepatoprotective effect of pre-treatment with licorice extract against CCl4-induced rat hepatic destruction evinced by decreased serum ALT, AST, and ALP activities. Salary et al. [2] reported that supplementation of 0.4% licorice extract in the drinking water of broiler chicks reduced ALT levels (*p* < 0.05). Licorice extract has many natural active compounds, including five flavonoids, glabridin (GLD), isoliquiritigenin (ISL), liquiritigenin (LTG), and licochalcone E (LCE), and three triterpenoids, 18α-glycyrrhizic acid (18α-GC), 18β-glycyrrhetinic acid (18β-GA), and 18β- glycyrrhizic acid (18β-GC), every one of them has an anti-diabetic effect via several mechanisms [57]. Wu et al. [58] showed that glabridin has hypoglycemic effects by modifying glucose and lipid metabolism. Our serum glucose concentration results showed the hypoglycemic effect of both licorice treatments at 0.4 and 0.8 g/L in the drinking water, agreeing with Moradi et al. [41]. They showed that administration of 0.2 and 0.3 g/L of licorice extract via drinking water lowers serum glucose concentration.

There are four mechanisms for reducing body fat, including decreasing calorie intake [59], inhibition of lipid absorption [60], decreasing fatty acids (FA) biosynthesis, and increasing fatty acid oxidation (FAO) [61]. In the present study, supplementation with licorice reduced triglyceride (TG) levels, indicating the suppression of lipid absorption effect, which reflected abdominal fat that decreased with increasing the licorice level. Interestingly, our serum results showed decreased TC, LDL-C, FFA, and increased HDL-C levels and HDL-C/LDL-C ratio. These results agree with [62], who reported that *Glycyrrhiza glabra* significantly decreased TC, LDL, and triglyceride (TG) levels, with an increase in high-HDL indicating its hypolipidemic effect. Moreover, Won et al. [63] reported that licorice is considered a food additive for obesity. Moradi et al. [41] reported that the serum TC and LDL-C concentrations decreased in chickens supplemented with 0.3 g/L of licorice extract via drinking water. Visavadiya and Narasimhacharya [45] showed that licorice extract has a cholesterol-lowering effect in rats owed to an elevation of cholesterol excretion, bile acid, neutral sterols, and increased bile acid content in the liver. Lipid peroxidation, lipoxygenase, and cyclooxygenase may be inhibited, which could explain this effect. The cholesterol-lowering effects of LE are attributed to the high secretion of cholesterol, bile acids, neutral sterols, and improvement in the content of hepatic bile acid. In addition, the active components of licorice (saponin) are able to reduce the levels of LDL-associated carotenoid [11].

Lysosomes are responsible for hydrolyzing complex macromolecules such as lipids, glycolipids, nucleic acid, complex sugars, and recycling cellular debris [64]. Lysozyme stimulates phagocytosis, and polymorphonuclear leucocytes increase non-specific immunity and resistance against diseases [38]. In the present study, licorice supplementation increased the serum lysosomal activity of broiler chicks in a dose-dependent manner (0.4 g/L and 0.8 g/L), indicating its immunostimulant effect. Similarly, Mishra et al. [65] reported that licorice has anti-arthritic activity by stabilizing lysosomal enzyme activity. The present study revealed that the addition of licorice in drinking water (0.4 g/L and 0.8 g/L) exhibits a broad range of biological activities such as hypolipidemic, hypoglycemic, hepatoprotective, immunostimulant, and antioxidant effects. The use of licorice up to 0.4 g/L in the drinking water of poultry increased the feed intake, improved the immune response and antioxidant parameters and lipid profile, and improved the cellular immunity, and may this explain our findings concerning body weight [13]. Our result concerning the IGF-1 and GHr has supported the growth performance traits. This result was supported by [66], which revealed that liquorice (*Glycyrrhiza glabra*) was utilized to increase feed efficiency. The phytobiotics’ anti-bacterial, immunomodulatory, antioxidative, and growth-promoting capabilities and their abundance of physiologically active chemicals may be to blame for the increased weight gain in the host [67]. In addition, to improve the growth of broiler chickens, *Glycyrrhiza glabra* serves two functions [3]; another may be due to its active ingredients, especially flavonoids, a pentacyclic triterpene, and glycyrrhizin [68]. Upregulation of the growth hormone and the hepatic growth hormone receptor, which increases the concentration of insulin-like growth factor 1, may explain the beneficial effect of *Glycyrrhiza glabra* flavonoids such as glabridin, liquiritin apioside, liquiritigenin, and isoliquiritoside on broiler growth performance [69]. Honda et al. [70] explored the molecular pathways by which licorice reduced fat storage and triglyceride in the liver, plasma, and VLDL. In addition, the enzymatic activities of ACC and FAS were significantly decreased by *Glycyrrhiza glabra*; It has been suggested that licorice reduces abdominal fat via regulating liver enzymes involved in the synthesis and oxidation of fatty acids, according to this new research [71]. The transcription factor for fatty acid oxidative enzymes is known as PPAR-α [72]. we studied the hepatic expression of the PPAR-α. Licorice augmented the hepatic PPAR-α mRNA level provided that the licorice PPAR-α regulates fatty acid oxidative enzymes. On the other hand, triglyceride-rich lipoproteins are mostly broken down by lipoprotein lipase (LPL). Apolipoprotein C-II enhances, and apolipoprotein C-III inhibits its activity. Apo C-II was activated, but Apo C-III was suppressed by licorice treatment. These indicated that GA promoted triglyceride metabolism by inducing LPL activity [73]. These findings supported our results in which licorice treatment significantly upregulated the mRNA LPL expression. Our results revealed that there were significant increases in the mRNA expression of the hepatic SOD1, and CAT genes in broilers fed licorice significantly (*p* < 0.05) increased compared to their control. This finding was consistent with [74] in which it was revealed that licorice contains bioactive compounds such as antioxidant flavonoids, which have anti-inflammatory and antioxidant properties [20,21,22,23]. It is thought that the antioxidant enzyme activities of licorice extract are increased since they reduce the oxidative rate and reactive substance formation in mitochondria [75].

## 5. Conclusions

Licorice improved broiler performance by influencing the molecular pathways that control growth and also antioxidant activity. It modulates the lipid metabolism genes. The licorice extract’s immunogenic and antioxidant properties may increase birds’ growth performance, feed efficiency, carcass traits, and biochemical blood indices. Increased feed intake, higher immunological response and antioxidant markers, and a better lipid profile were all seen when LE up to 0.4 g/L was added to chicken drinking water. Overall, the present work demonstrated that licorice inclusion in poultry diets could be used as a growth promoter; this study reports the molecular pathway by which the licorice supplementation achieved its action.

## Figures and Tables

**Table 1:** Ingredient composition and chemical analysis of the used experimental diets.

Ingredients	Starter	Grower	Finisher
Yellow corn (7.8% CP)	53	60	66
Soybean meal (44% CP)	35.4	29.7	24.2
Corn gluten (59.2% CP)	5.8	5.4	5.2
Vegetable oil ^1^	2	1.5	1.5
DCP ^2^	1.35	1	0.9
Lime-stone ^3^	1.6	1.6	1.4
Lysine ^4^	0.05	0.05	0.05
DL-Methionine ^5^	0.1	0.05	0.05
Choline ^6^	0.05	0.05	0.05
Mycotoxin adsorpant ^7^	0.05	0.05	0.05
Salt	0.3	0.3	0.3
Premix (mineral and vitamin) ^8^	0.3	0.3	0.3
Total	100	100	100
Chemical analysis:			
Moisture %	11.9	12.1	11.7
Crude Protein %	22.9	21.1	18.9
Ether Extract %	6.3	5.8	6.1
Ash %	5.8	5.6	5.9
ME kcal/kg diet *	3000	3053	3129

^1^ Mixture of sunflower and soybean oils. ^2^ Dicalcium phosphate: contains 21% calcium and 18.5% phosphorus. ^3^ Limestone contains 37% calcium and is locally produced. ^4^ Lysine 87% produced by ADM Co. Delaware. USA. ^5^ DL-methionine produced by Evonik Degussa GmbH, Hanau-Wolfgang, Germany. Guaranteed analysis 99.5% DL-methionine. ^6^ Choline: choline chloride 60% with the vegetable carrier (corn powder) produced by Shandyuong Pharmaceutical Co. China. ^7^ Beta-2-x. ^8^ Each 3 kg contains: Vit. A (12,000,000 IU), Vit. D (2,000,000 IU), Vit. E (10 g), Vit. K3 (2 g), Vit. B1 (1 g), Vit. B2 (5 g), Vit. B6 (1.5 g), Vit. B12 (10 g), Nicotinic acid (30 g), Pantothenic acid (10 g), Folic acid (1 g), Biotin (50 mg), Choline chloride 50% (250 mg), Iron (30 g), Copper (10 g), Zinc (50 g), Manages (60 g), Iodine (1 g), Selenium (0.1 g), Cobalt (0.1 g) and carrier limestone up to 3 kg. * The feed composition calculated according to NRC (1994) [22].

**Table 2:** Primers for gene expression by RT-PCR.

Gene	Direction	Primer Sequence	Pb	Accession Number
**SOD1**	Sense	CACTGCATCATTGGCCGTACCA	226	NM_205064.1
	Antisense	GCTTGCACACGGAAGAGCAAGT		
**CAT**	Sense	TGGCGGTAGGAGTCTGGTCT	115	NM_001031215.1
	Antisense	GTCCCGTCCGTCAGCCATTT		
**IGF1**	Sense	CACCTAAATCTGCACGCT	142	NM_001004384.3
	Antisense	CTTGTGGATGGCATGATCT		
**GHr**	Sense	AACACAGATACCCAACAGCC	145	AH007376.2
	Antisense	AGAAGTCAGTGTTTGTCAGGG		
**PPARα**	Sense	ACGAATGCCAAGGTCTGAGA	170	NM_001001464.1
	Antisense	TGCAAGGATGACTCTGGCTT		
**FAS**	Sense	TGGTTGACTGCCACCAATTG	215	J04485.1
	Antisense	ACCCCACTTTCCATCACGAT		
**LPL**	Sense	GGATTGCTGGAAGTTTAACCAAG	330	NM_205282.2
	Antisense	AGAGATGGATGGATCGTTCATGA		
**Β.actin**	Sense	AGCGAACGCCCCCAAAGTTCT	139	NM_205518.1
	Antisense	AGCTGGGCTGTTGCCTTCACA		

CAT, Catalase; SOD1, Superoxidismutase1. IGF-1, insulin growth factor-1. GHr, Growth hormone receptor. PPARα, Peroxisome proliferator-activated receptor. FAS, Fatty acid synthase. LPL, lipoprotein lipase.

**Table 3:** Effect of licorice supplementation in drinking water on body weight, weight gain, feed intake, and feed conversion ratio of broiler chicken.

Variable	Treatment Groups			*p*-Value
	Group 1 (Control)	Group 2 (Licorice 0.4 gm/L)	Group 3 (Licorice 0.8 gm/L)	
Initial weight, week 1	180.95 ± 2.11	181.60 ± 1.06	181.87 ± 1.42	NS
Week 2	439.62 ± 5.29 ^b^	478.07 ± 4.18 ^a^	465.47 ± 6.14 ^a^	<0.0001
Week 3	872.93 ± 9.02 ^b^	963.36 ± 7.66 ^a^	944.40 ± 14.69 ^a^	<0.0001
Week 4	1533.31 ± 16.67 ^b^	1624.29 ± 13.98 ^a^	1546.20 ± 25.35 ^b^	0.0019
Week 5	2078.57 ± 22.49 ^c^	2360.57 ± 29.57 ^a^	2230.13 ± 27.13 ^b^	<0.0001
Final Weight, Week 6	2573.43 ± 23.29 ^b^	2879.93 ± 36.06 ^a^	2652.40 ± 35.29 ^b^	<0.0001
Gain1, W1–W2	258.67 ± 3.39 ^c^	296.13 ± 3.40 ^a^	283.60 ± 5.49 ^b^	<0.0001
Gain2, W2–W3	433.31 ± 3.94 ^b^	485.29 ± 4.77 ^a^	478.93 ± 9.01 ^a^	<0.0001
Gain3, W3–W4	660.38 ± 8.47 ^a^	660.93 ± 6.67 ^a^	601.80 ± 12.05 ^b^	<0.0001
Gain4, W4–W5	545.26 ± 8.15 ^c^	736.29 ± 17.29 ^a^	683.93 ± 5.51 ^b^	<0.0001
Gain5, W5–W6	494.86 ± 17.80 ^a^	519.36 ± 8.74 ^a^	422.27 ± 10.08 ^b^	<0.0001
TGain, W1–W6	2392.48 ± 22.32 ^b^	2697.99 ± 35.13 ^a^	2470.53 ± 34.40 ^b^	<0.0001
FI1	428.6 ± 0.00 ^b^	424.20 ± 0.00 ^c^	436.70 ± 0.00 ^a^	<0.0001
FI2	626.37 ± 0.00 ^c^	703.20 ± 0.00 ^a^	696.80 ± 0.00 ^b^	<0.0001
FI3	969.06 ± 0.00 ^b^	1018.50 ± 0.00 ^a^	969.06 ± 0.00 ^b^	<0.0001
FI4	1148.00 ± 0.00 ^c^	1329.00 ± 0.00 ^a^	1290.00 ± 0.00 ^b^	<0.0001
FI5	1245.10 ± 0.00 ^b^	1219.20 ± 0.00 ^c^	1307.70 ± 0.00 ^a^	<0.0001
TFI	4417.13 ± 0.00 ^b^	4694.10 ± 0.00 ^a^	4700.26 ± 0.00 ^a^	<0.0001
FCR1	1.66 ± 0.02 ^a^	1.44 ± 0.02 ^c^	1.57 ± 0.03 ^b^	<0.0001
FCR2	1.45 ± 0.01	1.46 ± 0.02	1.48 ± 0.02	NS
FCR3	1.48 ± 0.02 ^c^	1.55 ± 0.02 ^b^	1.65 ± 0.03 ^a^	<0.0001
FCR4	2.13 ± 0.03 ^a^	1.88 ± 0.05 ^b^	1.89 ± 0.02 ^b^	<0.0001
FCR5	2.50 ± 0.06 ^b^	2.40 ± 0.05 ^b^	3.19 ± 0.07 ^a^	<0.0001
FCR	1.84 ± 0.02 ^b^	1.74 ± 0.02 ^c^	1.90 ± 0.03 ^a^	<0.0001

The data presented in the table are mean ± SEM. Those within a row superscripted by different letters are significantly different (*p* < 0.05). NS: non-significant. TGain: Total gain; FCR: Feed conversion ratio. FI1: Feed intake for the first week; TFI: total feed intake.

**Table 4:** Effect of licorice supplementation in drinking water on some organs relative to slaughter weight of broiler chicken.

Variable	Treatment Groups			*p*-Value
	Group 1 (Control)	Group 2 (Licorice 0.4 gm/L)	Group 3 (Licorice 0.8 gm/L)	
Liver	2.32 ± 0.02	2.33 ± 0.07	2.43 ± 0.05	NS
Gizzard	0.95 ± 0.08	1.02 ± 0.03	0.99 ± 0.02	NS
Proventriculous	0.32 ± 0.01	0.29 ± 0.02	0.32 ± 0.02	NS
Heart	0.41 ± 0.01	0.45 ± 0.03	0.41 ± 0.03	NS
Abdominal fat	1.30 ± 0.06 ^a^	1.00 ± 0.17 ^ab^	0.78 ± 0.05 ^b^	0.008
Spleen	0.13 ± 0.01	0.10 ± 0.01	0.14 ± 0.02	NS
Bursa of Fabricius	0.18 ± 0.01 ^b^	0.41 ± 0.11 ^a^	0.13 ± 0.01 ^b^	0.01
Thymus	0.34 ± 0.02	0.24 ± 0.01	0.29 ± 0.04	NS
Intestine	4.05 ± 0.09	3.98 ± 0.30	3.67 ± 0.07	NS

The data presented in the table are mean ± SEM. Those within a row superscripted by different letters are significantly different (*p* < 0.05)—NS: non-significant.

**Table 5:** Effect of licorice supplementation in drinking water on serum lipid profile of broiler chicken.

Variable	Treatment Groups			*p*-Value
	Group 1 (Control)	Group 2 (Licorice 0.4 gm/L)	Group 3 (Licorice 0.8 gm/L)	
TC (mg/dL)	101.00 ± 4.44 ^a^	74.00 ± 3.33 ^b^	73.67 ± 2.05 ^b^	<0.0001
TG (mg/dL)	44.00 ± 1.73 ^a^	26.33 ± 1.20 ^b^	28.67 ± 1.45 ^b^	<0.0001
HDL (mg/dL)	9.47 ± 0.27 ^b^	14.23 ± 1.30 ^a^	14.73 ± 0.45 ^a^	0.0002
LDL (mg/dL)	82.73 ± 4.49 ^a^	54.50 ± 2.59 ^b^	53.20 ± 2.58 ^b^	<0.0001
HDL/LDL Ratio	0.12 ± 0.1 ^b^	0.26 ± 0.02 ^a^	0.29 ± 0.02 ^a^	<0.0001
FFA	10.37 ± 0.21 ^a^	7.63 ± 0.28 ^b^	7.83 ± 0.53 ^b^	<0.0001

The data presented in the table are mean ± SEM. Those within a row superscripted by different letters are significantly different (*p* < 0.05). Total cholesterol (TC), low-density lipoprotein cholesterol (LDL), High-density lipoprotein (HDL). free fatty acid (FFA), and triglyceride (TG).

**Table 6:** Effect of licorice supplementation in drinking water on serum liver biomarker and serum glucose level of broiler chicken.

Variable	Treatment Groups			*p*-Value
	Group 1 (Control)	Group 2 (Licorice 0.4 gm/L)	Group 3 (Licorice 0.8 gm/L)	
Protein (g/L)	3.10 ± 0.26	2.77 ± 0.06	3.13 ± 0.03	NS
Albumen (g/L)	2.36 ± 0.11 ^a^	2.08 ± 0.03 ^b^	2.13 ± 0.03 ^b^	0.0171
Globulin (g/L)	0.74 ± 0.29	0.69 ± 0.09	1.00 ± 0.00	NS
ALT (U/L)	37.67 ± 1.20 ^a^	27.67 ± 0.93 ^b^	22.00 ± 1.04 ^c^	<0.0001
AST (U/L)	47.67 ± 1.76 ^a^	34.00 ± 2.52 ^b^	34.67 ± 1.69 ^b^	<0.0001
Glucose (mg/dl)	98.67 ± 3.06 ^a^	75.33 ± 2.24 ^b^	77.00 ± 3.50 ^b^	<0.0001

The data presented in the table are mean ± SEM. Those within a row superscripted by different letters are significantly different (*p* < 0.05)—NS: non-significant. ALT: alanine aminotransferase. AST: aspartate aminotransferase.

**Table 7:** Effect of licorice supplementation in drinking water on oxidative stress, immunostimulant biomarkers of broiler chicken.

Variable	Treatment Groups			*p*-Value
	Group 1 (Control)	Group 2 (Licorice 0.4 gm/L)	Group 3 (Licorice 0.8 gm/L)	
GSH (mg/dL)	40.33 ± 1.76 ^b^	48.67 ± 1.01 ^a^	50.67 ± 3.28 ^a^	0.0075
MDA (nmol/mL)	5.40 ± 0.16 ^a^	3.77 ± 0.12 ^b^	3.60 ± 0.22 ^b^	<0.0001
Catalase (U/L)	35.33 ± 1.30 ^b^	42.00 ± 0.87 ^a^	42.67 ± 1.74 ^a^	0.0012
Lysozyme	86.33 ± 0.60 ^c^	92.00 ± 1.26 ^b^	103.33 ± 2.46 ^a^	<0.0001

The data presented in the table are mean ± SEM. Those within a row superscripted by different letters are significantly different (*p* < 0.05).

**Table 8:** Morphometric parameters of duodenum and ileum of broiler chickens supplemented with licorice with different concentrations.

	Control	Licorice 0.4 g/L	Licorice 0.8 g/L
Duodenum			
villi height	486.5 ± 8.5 ^b^	556.8 ± 9.45 ^a^	461.5 ± 14.5 ^c^
crypt depth	73.3 ± 5.2 ^b^	64.6 ± 5.6 ^c^	88.1 ± 7.1 ^a^
villi height/crypt depth	6.9 ± 0.45 ^b^	9 ± 0.25 ^a^	4.9 ± 0.47 ^c^
villi width	67.3 ± 2.14 ^b^	80.7 ± 4.1 ^a^	57.8 ± 2.1 ^c^
Ileum			
villi height	285.396 ± 5.14 ^b^	290.12 ± 9.1 ^a^	278.9 ± 12.14 ^c^
crypt depth	41.766 ± 4.6 ^a^	31.2 ± 1.45 ^b^	40.14 ± 3.1 ^a^
villi height/crypt depth	6.8332 ± 0.78 ^b^	9.159 ± 0.78 ^a^	6.948 ± 0.78 ^b^
villi width	44.02 ± 3.1 ^b^	56.76 ± 3.6 ^a^	57.96 ± 3.4 ^a^

The data presented in the table are mean ± SEM. Those within a row superscripted by different letters are significantly different (*p* < 0.05).

## Data Availability

Data are available upon request.

## References

[1] Da Costa P.M., Bica A., Vaz-Pires P., Bernardo F. (2010). Changes in antimicrobial resistance among faecal enterococci isolated from growing broilers prophylactically medicated with three commercial antimicrobials. Prev. Vet. Med..

[2] Salary J., Kalantar M., Ala M., Ranjbar K., Matin H.H. (2014). Drinking water supplementation of licorice and aloe vera extracts in broiler chickens. Sci. J. Anim. Sci..

[3] Alagawany M., Elnesr S.S., Farag M.R., Abd El-Hack M.E., Khafaga A.F., Taha A.E., Tiwari R., Yatoo M., Bhatt P. (2019). Use of licorice (*Glycyrrhiza glabra*) herb as a feed additive in poultry: Current knowledge and prospects. Animals.

[4] Shibata S. (2000). A drug over the millennia: Pharmacognosy, chemistry, and pharmacology of licorice. Yakugaku Zasshi.

[5] Dastagir G., Rizvi M.A. (2016). Glycyrrhiza glabra L. (Liquorice). Pak. J. Pharm. Sci..

[6] Pastorino G., Cornara L., Soares S., Rodrigues F., Oliveira M.B.P. (2018). Liquorice (*Glycyrrhiza glabra*): A phytochemical and pharmacological review. Phytother. Res..

[7] Karahan F., Avsar C., Ozyigit I.I., Berber I. (2016). Antimicrobial and antioxidant activities of medicinal plant *Glycyrrhiza glabra* var. glandulifera from different habitats. Biotechnol. Biotechnol. Equip..

[8] Fiore C., Eisenhut M., Krausse R., Ragazzi E., Pellati D., Armanini D., Bielenberg J. (2008). Antiviral effects of Glycyrrhiza species. Phytother. Res. Int. J. Devoted Pharmacol. Toxicol. Eval. Nat. Prod. Deriv..

[9] Somjen D., Knoll E., Vaya J., Stern N., Tamir S. (2004). Estrogen-like activity of licorice root constituents: Glabridin and glabrene, in vascular tissues in vitro and in vivo. J. Steroid Biochem. Mol. Biol..

[10] Alagawany M., Abd El-Hack M.E., Farag M.R., Elnesr S.S., El-Kholy M.S., Saadeldin I.M., Swelum A.A. (2018). Dietary supplementation of *Yucca schidigera* extract enhances productive and reproductive performances, blood profile, immune function, and antioxidant status in laying Japanese quails exposed to lead in the diet. Poult. Sci..

[11] Sedghi M., Golian A., Kermanshahi H., Ahmadi H. (2011). Effect of dietary supplementation of licorice extract and a prebiotic on performance and blood metabolites of broilers. S. Afr. J. Anim. Sci..

[12] Ibrahim D., Sewid A.H., Arisha A.H., El-Fattah A.H.A., Abdelaziz A.M., Al-Jabr O.A., Kishawy A.T.Y. (2020). Influence of Glycyrrhiza glabra Extract on Growth, Gene Expression of Gut Integrity, and Campylobacter jejuni Colonization in Broiler Chickens. Front. Vet. Sci..

[13] Alagawany M., Elnesr S., Farag M. (2019). Use of liquorice (*Glycyrrhiza glabra*) in poultry nutrition: Global impacts on performance, carcass and meat quality. World’s Poult. Sci. J..

[14] Jagadeeswaran A., Selvasubramanian S. (2014). Effect of supplementation of licorice root (*Glycyrrhiza glabra* L.) extracts on immune status in commercial broilers. Int. J. Adv. Vet. Sci. Technol.

[15] Rashidi N., Ghorbani M.R., Tatar A., Salari S. (2019). Response of broiler chickens reared at high density to dietary supplementation with licorice extract and probiotic. J. Anim. Physiol. Anim. Nutr..

[16] Varsha S., Agrawal R., Sonam P. (2013). Phytochemical screening and determination of anti-bacterial and anti-oxidant potential of *Glycyrrhiza glabra* root extracts. J. Environ. Res. Dev..

[17] Sohail M., Rakha A., Butt M.S., Asghar M. (2018). Investigating the antioxidant potential of licorice extracts obtained through different extraction modes. J. Food Biochem..

[18] Thakur A., Raj P. (2017). Pharmacological perspective of *Glycyrrhiza glabra* Linn: A mini-review. J. Anal. Pharm. Res.

[19] Badr S.E., Sakr D.M., Mahfouz S.A., Abdelfattah M.S. (2013). Licorice (*Glycyrrhiza glabra* L.): Chemical composition and biological impacts. Res. J. Pharm. Biol. Chem. Sci..

[20] Shabani L., Ehsanpour A., Asghari G., Emami J. (2009). Glycyrrhizin production by in vitro cultured *Glycyrrhiza glabra* elicited by methyl jasmonate and salicylic acid. Russ. J. Plant Physiol..

[21] Beski S., Shekhu N., Sadeq S., Al-Khdri A., Ramadhan N., AL-Bayati S. (2019). Effects of the Addition of Aqueous Liquorice (*Glycyrrhiza glabra*) Extract to Drinking Water in the Production Performance, Carcass Cuts and Intestinal Histomorphology Of Broiler Chickens. Iraqi J. Agric. Sci..

[22] NRC (1994). Nutrient Requirements of Poultry.

[23] AOAC (1990). Official Methods of Analysis.

[24] Randhir S., Pradhan K. (1981). Forage Evaluation. First Published, Printox, New Dalhi.

[25] Hanson S. (1963). Application of the Bligh and Dyer method of lipid extraction to tissue homogenates. J. Biochem..

[26] Allain C.C., Poon L.S., Chan C.S., Richmond W., Fu P.C. (1974). Enzymatic determination of total serum cholesterol. Clin. Chem..

[27] Vassault A., Grafmeyer D., Naudin C., Dumont G., Bailly M., Henny J., Gerhardt M., Georges P. (1986). Protocole de validation de techniques. Ann Biol Clin..

[28] McGowan M.W., Artiss J.D., Strandbergh D.R., Zak B. (1983). A peroxidase-coupled method for the colorimetric determination of serum triglycerides. Clin. Chem..

[29] Swelum A.A., Shafi M.E., Albaqami N.M., El-Saadony M.T., Elsify A., Abdo M., Taha A.E., Abdel-Moneim A.E., Al-Gabri N.A., Almaiman A.A. (2020). COVID-19 in human, animal, and environment: A review. Front. Vet. Sci..

[30] Songserm T., Engel B., Van Roozelaar D., Kok G., Pijpers A., Pol J., Ter Huurne A. (2002). Cellular immune response in the small intestine of two broiler chicken lines orally inoculated with malabsorption syndrome homogenates. Vet. Immunol. Immunopathol..

[31] Der G., Everitt B.S. (2008). A Handbook of Statistical Analyses Using SAS.

[32] Dhama K., Latheef S.K., Mani S., Samad H.A., Karthik K., Tiwari R., Khan R.U., Alagawany M., Farag M.R., Alam G.M. (2015). Multiple beneficial applications and modes of action of herbs in poultry health and production-A review. Int. J. Pharmacol..

[33] Upadhayay U., Vishwa P.C.V. (2014). Growth promoters and novel feed additives improving poultry production and health, bioactive principles and beneficial applications: The trends and advances-a review. Int. J. Pharm..

[34] Myandoab M.P., Hosseini Mansoub N. (2012). Comparative effect of Liquorice root extract medicinal plants and probiotic in diets on performance, carcass traits and serum composition of Japanese quails. Glob. Vet..

[35] Al-Zuhairy M., Hashim M.E. (2015). Influence of different levels of licorice (Glycyrrhizaglabra Inn.) and garlic (Alliumsativum) mixture powders supplemented diet on broiler Productive traits. Iraqi J. Vet. Med..

[36] Lashin I., Iborahem I., Ola F., Talkhan F., Mohamed F. (2017). Influence of licorice extract on heat stress in broiler chickens. Anim. Health Res. J..

[37] Ocampo C., Gómez-Verduzco G., Tapia-Perez G., Gutierrez O., Sumano L. (2016). Effects of glycyrrhizic acid on productive and immune parameters of broilers. Braz. J. Poult. Sci..

[38] Alagawany M., Farag M.R., Salah A.S., Mahmoud M.A. (2020). The role of oregano herb and its derivatives as immunomodulators in fish. Rev. Aquac..

[39] Al-Daraji H.J. (2012). Influence of drinking water supplementation with licorice extract on certain blood traits of broiler chickens during heat stress. Pharmacogn. Commun..

[40] Hosseini S., Goudarzi M., Zarei A., Meimandipour A., Sadeghipanah A. (2014). The effects of funnel and licorice on immune response, blood parameter and gastrointestinal organs in broiler chiks. Iran. J. Med. Aromat. Plants.

[41] Moradi N., Ghazi S., Amjadian T., Khamisabadi H., Habibian M. (2014). Performance and Some Immunological Parameter Responses of Broiler Chickens to Licorice (Glycyrrhiza glabra) Extract Administration in the Drinking Water. Annu. Res. Rev. Biol..

[42] Bown D. (1995). Encyclopedia of Herbs & Their Uses.

[43] Aoki F., Honda S., Kishida H., Kitano M., Arai N., Tanaka H., Yokota S., Nakagawa K., Asakura T., Nakai Y. (2007). Suppression by licorice flavonoids of abdominal fat accumulation and body weight gain in high-fat diet-induced obese C57BL/6J mice. Biosci. Biotechnol. Biochem..

[44] Tominaga Y., Mae T., Kitano M., Sakamoto Y., Ikematsu H., Nakagawa K. (2006). Licorice flavonoid oil effects body weight loss by reduction of body fat mass in overweight subjects. J. Health Sci..

[45] Visavadiya N.P., Narasimhacharya A.V. (2006). Hypocholesterolaemic and antioxidant effects of *Glycyrrhiza glabra* (Linn) in rats. Mol. Nutr. Food Res..

[46] Jung J.-C., Lee Y.-H., Kim S.H., Kim K.-J., Kim K.-M., Oh S., Jung Y.-S. (2015). Hepatoprotective effect of licorice, the root of Glycyrrhiza uralensis Fischer, in alcohol-induced fatty liver disease. BMC Complementary Altern. Med..

[47] Kwon H.-M., Choi Y.-J., Choi J.-S., Kang S.-W., Bae J.-Y., Kang I.-J., Jun J.-G., Lee S.-S., Lim S.S., Kang Y.-H. (2007). Blockade of cytokine-induced endothelial cell adhesion molecule expression by licorice isoliquiritigenin through NF-κB signal disruption. Exp. Biol. Med..

[48] Reda F.M., El-Saadony M.T., El-Rayes T.K., Farahat M., Attia G., Alagawany M. (2021). Dietary effect of licorice (*Glycyrrhiza glabra*) on quail performance, carcass, blood metabolites and intestinal microbiota. Poult. Sci..

[49] Vaillancourt K., LeBel G., Pellerin G., Ben Lagha A., Grenier D. (2021). Effects of the licorice isoflavans licoricidin and glabridin on the growth, adherence properties, and acid production of Streptococcus mutans, and assessment of their biocompatibility. Antibiotics.

[50] de Paiva G.B., Trindade M.A., Romero J.T., da Silva-Barretto A.C. (2021). Antioxidant effect of acerola fruit powder, rosemary and licorice extract in caiman meat nuggets containing mechanically separated caiman meat. Meat Sci..

[51] Rashidi N., Khatibjoo A., Taherpour K., Akbari-Gharaei M., Shirzadi H. (2020). Effects of licorice extract, probiotic, toxin binder and poultry litter biochar on performance, immune function, blood indices and liver histopathology of broilers exposed to aflatoxin-B1. Poult. Sci..

[52] Dosoky W.M., Zeweil H.S., Ahmed M.H., Zahran S.M., Ali A.M., Abdelsalam N.R., Naiel M.A.E. (2021). The influences of Tylosine and licorice dietary supplementation in terms of the productive performance, serum parameters, egg yolk lipid profile, antioxidant and immunity status of laying Japanese quail under heat stress condition. J. Therm. Biol..

[53] Hashem M.A., Abdallah A.A., Eldeen I.G., Amer M.M. (2017). Biochemical Studies on Rosemary and Licorice against Lead-Induced Oxidative Stress in Rats. Zagazig Vet. J..

[54] Habibi R., Sadeghi G., Karimi A. (2014). Effect of different concentrations of ginger root powder and its essential oil on growth performance, serum metabolites and antioxidant status in broiler chicks under heat stress. Br. Poult. Sci..

[55] Sen S., Roy M., Chakraborti A.S. (2011). Ameliorative effects of glycyrrhizin on streptozotocin-induced diabetes in rats. J. Pharm. Pharmacol..

[56] Huo H.Z., Wang B., Liang Y.K., Bao Y.Y., Gu Y. (2011). Hepatoprotective and antioxidant effects of licorice extract against CCl4-induced oxidative damage in rats. Int. J. Mol. Sci..

[57] Yang L., Jiang Y., Zhang Z., Hou J., Tian S., Liu Y. (2020). The anti-diabetic activity of licorice, a widely used Chinese herb. J. Ethnopharmacol..

[58] Wu F., Jin Z., Jin J. (2013). Hypoglycemic effects of glabridin, a polyphenolic flavonoid from licorice, in an animal model of diabetes mellitus. Mol. Med. Rep..

[59] Van Gaal L.F., Rissanen A.M., Scheen A.J., Ziegler O., Rössner S., Group R.-E.S. (2005). Effects of the cannabinoid-1 receptor blocker rimonabant on weight reduction and cardiovascular risk factors in overweight patients: 1-year experience from the RIO-Europe study. Lancet.

[60] Nakai M., Fukui Y., Asami S., Toyoda-Ono Y., Iwashita T., Shibata H., Mitsunaga T., Hashimoto F., Kiso Y. (2005). Inhibitory effects of oolong tea polyphenols on pancreatic lipase in vitro. J. Agric. Food Chem..

[61] Murase T., Nagasawa A., Hase T., Tokimitsu I., Shimasaki H., Itakura H. (2001). Dietary tea catechins reduce development of obesity accompanied with gene expression of lipid-metabolizing enzymes in mice. J. Oleo Sci..

[62] Alwash Y.S., Latif A.R.A., Al-Bayati N.J. (2011). Effect of licorice extract on lipid profile in hypercholestermic male rabbits. Al-Qadisiyah Med. J..

[63] Won S.-R., Kim S.-K., Kim Y.-M., Lee P.-H., Ryu J.-H., Kim J.-W., Rhee H.-I. (2007). Licochalcone A: A lipase inhibitor from the roots of Glycyrrhiza uralensis. Food Res. Int..

[64] Yu C., Sun Q., Zhou H. (2013). Enzymatic screening and diagnosis of lysosomal storage diseases. N. Am. J. Med. Sci..

[65] Mishra N., Bstia S., Mishra G., Chowdary K., Patra S. (2011). Anti-arthritic activity of *Glycyrrhiza glabra*, Boswellia serrata and their synergistic activity in combined formulation studied in freund’s adjuvant induced arthritic rats. J. Pharm. Educ. Res..

[66] Gilani S.M.H., Rashid Z., Galani S., Ilyas S., Sahar S., Al-Ghanim K., Zehra S., Azhar A., Al-Misned F., Ahmed Z. (2021). Growth performance, intestinal histomorphology, gut microflora and ghrelin gene expression analysis of broiler by supplementing natural growth promoters: A nutrigenomics approach. Saudi J. Biol. Sci..

[67] Reis J.H., Gebert R.R., Barreta M., Baldissera M.D., Dos Santos I.D., Wagner R., Campigotto G., Jaguezeski A.M., Gris A., de Lima J.L. (2018). Effects of phytogenic feed additive based on thymol, carvacrol and cinnamic aldehyde on body weight, blood parameters and environmental bacteria in broilers chickens. Microb. Pathog..

[68] Kim S.C., Byun S.H., Yang C.H., Kim C.Y., Kim J.W., Kim S.G. (2004). Cytoprotective effects of Glycyrrhizae radix extract and its active component liquiritigenin against cadmium-induced toxicity (effects on bad translocation and cytochrome c-mediated PARP cleavage). Toxicology.

[69] Ouyang K., Xu M., Jiang Y., Wang W. (2016). Effects of alfalfa flavonoids on broiler performance, meat quality, and gene expression. Can. J. Anim. Sci..

[70] Honda K., Kamisoyama H., Tominaga Y., Yokota S., Hasegawa S. (2009). The molecular mechanism underlying the reduction in abdominal fat accumulation by licorice flavonoid oil in high fat diet-induced obese rats. Anim. Sci. J..

[71] Kamisoyama H., Honda K., Tominaga Y., Yokota S., Hasegawa S. (2008). Investigation of the anti-obesity action of licorice flavonoid oil in diet-induced obese rats. Biosci. Biotechnol. Biochem..

[72] Mandard S., Müller M., Kersten S. (2004). Peroxisome proliferator-activated receptor α target genes. Cell. Mol. Life Sci. CMLS.

[73] Wang C., Duan X., Sun X., Liu Z., Sun P., Yang X., Sun H., Liu K., Meng Q. (2016). Protective effects of glycyrrhizic acid from edible botanical *Glycyrrhiza glabra* against non-alcoholic steatohepatitis in mice. Food Funct..

[74] Vlaisavljević S., Šibul F., Sinka I., Zupko I., Ocsovszki I., Jovanović-Šanta S. (2018). Chemical composition, antioxidant and anticancer activity of licorice from Fruska Gora locality. Ind. Crops Prod..

[75] Yatoo M., Gopalakrishnan A., Saxena A., Parray O.R., Tufani N.A., Chakraborty S., Tiwari R., Dhama K., Iqbal H. (2018). Anti-inflammatory drugs and herbs with special emphasis on herbal medicines for countering inflammatory diseases and disorders-a review. Recent Pat. Inflamm. Allergy Drug Discov..

